# The Non-Traditional Cardiovascular Culprits in Chronic Kidney Disease: Mineral Imbalance and Uremic Toxin Accumulation

**DOI:** 10.3390/ijms26167938

**Published:** 2025-08-17

**Authors:** Yue Lu, Linlin Meng, Xinlu Wang, Yun Zhang, Cheng Zhang, Meng Zhang

**Affiliations:** State Key Laboratory for Innovation and Transformation of Luobing Theory, Key Laboratory of Cardiovascular Remodeling and Function Research of MOE, NHC, CAMS and Shandong Province, Department of Cardiology, Qilu Hospital of Shandong University, Cheeloo College of Medicine, Shandong University, Jinan 250012, China

**Keywords:** chronic kidney disease, cardiovascular disease, mineral homeostasis, uremic toxins, metabolic dysregulation

## Abstract

Chronic kidney disease (CKD) is associated with a significantly elevated mortality rate, primarily due to cardiovascular disease (CVD), highlighting a complex bidirectional relationship between the two conditions. Life-threatening cardiovascular events occur despite control of the traditional risk factors, emphasizing the underlying role of non-traditional risk factors. CKD, causing mineral imbalance and the accumulation of uremic toxins due to a compromised ability to excrete waste products, imposes extra pressure on the cardiovascular system. The retention of mineral and uremic toxins, in turn, aggravates the progression of CKD. This review aims to elucidate the pathophysiological connections between CKD and CVD, with a particular focus on the metabolic regulatory mechanisms influenced by minerals such as calcium and phosphate, as well as uremic toxins. We review how these factors contributed to accelerated multi-organ damage through mechanisms such as inflammation, endothelial dysfunction, oxidative stress, and vascular calcification. In addition, we discuss the therapeutic strategies for specific uremic toxins and proposed directions for future investigations. This review provides insights into the complex interplay between metabolic dysregulation and cardiovascular outcomes in CKD patients, promoting the development of innovative therapeutic interventions, ultimately improving the prognosis and quality of life for patients affected by these interconnected conditions.

## 1. Introduction

Chronic kidney disease (CKD) is defined as abnormalities in kidney structure or function lasting at least three months. According to the Kidney Disease: Improving Global Outcomes (KDIGO) guidelines [1], CKD is staged (1–5) by glomerular filtration rate (GFR) and albuminuria categories. The disease may begin at stage 1, where GFR is normal but damage is present, and advance to stages with progressively reduced renal function. The presence of albuminuria or other kidney damage markers defines CKD even in patients with GFR ≥ 60 mL/min/1.73 m^2^. Patients reaching stage 5 (GFR < 15 mL/min/1.73 m^2^), or end-stage renal disease, require dialysis or kidney transplantation to survive.

CKD has become a significant public health challenge, affecting approximately 15–20% of the global population and resulting in 5–10 million deaths annually [2,3]. Mortality among patients with CKD is predominantly attributed to the substantial burden of CVD and the elevated risk of major adverse cardiovascular events, including atherothrombotic disorders leading to myocardial infarction and stroke, heart failure, aortic disease, arrhythmias, and sudden cardiac death [2]. The current guideline-directed medical therapy for CVD events already comprises strict interventions on traditional risk factors, including smoking cessation, anti-hypertension, glycemic control, and aggressive reduction of cholesterol. However, life-threatening cardiovascular events occur despite control of the traditional risk factors, which are probably the consequences of residual cardiovascular risk [4]. CKD, causing volume overload, malnutrition, anemia, albuminuria, and accumulation of uremic toxins in plasma, imposes additional pressure on the cardiovascular system beyond the traditional risk factors, compared to non-CKD. A meta-analysis involving 1.4 million individuals from 30 published cohort studies indicated that, after adjustment for traditional cardiovascular risk factors, a risk gradient for cardiovascular mortality was apparent even in the early stages of CKD and increased linearly with CKD progression and the presence of albuminuria, suggesting that CKD has become an independent risk factor for CVD [5]. Therefore, targeting conventional CVD risk factors is insufficient to effectively prevent cardiovascular events in patients with CKD [6]. Focusing on non-traditional CKD-related risk factors could be a promising strategy for reducing the residual cardiovascular risk in CKD patients.

The underlying mechanisms linking CKD and CVD are multifactorial, involving shared risk factors such as hypertension, hyperglycemia, and dyslipidemia, as well as unique pathophysiological processes, including mineral imbalance and the accumulation of uremic toxins. In CKD, the renal ability to excrete waste products is compromised, leading to the retention of toxic metabolites and metabolic abnormalities of minerals that can adversely affect cardiovascular function. In addition, the accumulation of minerals and uremic toxins promotes renal impairment, further aggravating the severity of CKD. The identification and characterization of these toxins have become crucial in elucidating the pathophysiological links between CKD and CVD. This review aims to systematically explore the pathophysiological effects and potential therapeutic strategies of both minerals and uremic toxins, providing new insights into the complex interplay between CKD and CVD [7].

## 2. Mineral Metabolism

### 2.1. Dysregulation of Phosphate Homeostasis in CKD

Phosphate is essential for cellular and systemic homeostasis, involved in ATP synthesis, pH balance regulation, intracellular signaling, and as a major building block for bone, phospholipids, and nucleic acids. Within the human body, approximately 80–85% of phosphate resides in bone, with the remaining 15–20% in soft tissues and blood [7]. Dietary intake is the major source of phosphate, which is especially abundant in meat, fish, and dairy products. Phosphate absorption in the gastrointestinal tract primarily occurs through two distinct mechanisms: a passive paracellular pathway moving between enterocytes via tight junctions, and an active transcellular route mediated chiefly by the sodium-dependent phosphate transporter NaPi-IIb [8,9]. Under normal conditions, phosphate elimination is accomplished via glomerular filtration-mediated renal excretion, of which 80–90% is reabsorbed in the renal proximal tubules through the sodium-dependent transporter NaPi-IIa [10]. Consequently, phosphate output decreases as GFR declines in kidney failure. In the case of phosphate overload, NaPi-IIa transporters undergo endocytosis from the apical membranes of proximal tubule cells. This increases phosphate flow through the proximal nephron segment, elevating single-nephron fractional excretion of phosphate and thereby promoting phosphaturia. Besides the kidneys, bone is also a phosphate regulator via bone formation and resorption.

Multiple hormones coordinate systemic phosphate balance, including parathyroid hormone (PTH), fibroblast growth factor 23 (FGF-23), klotho, and active vitamin D [11]. These hormones function mainly by regulating intestinal absorption and renal reabsorption of phosphate. 1,25-dihydroxyvitamin D enhances intestinal phosphate uptake by upregulating the NaPi-IIb cotransporter [12]. Conversely, both PTH and FGF-23 decrease renal phosphate reabsorption and induce phosphaturia by triggering NaPi-IIa internalization and inactivation. Klotho serves as an essential cofactor for FGF-23 signaling but also independently suppresses NaPi-IIa activity to promote urinary phosphate excretion. Collectively, these mechanisms maintain serum phosphate within a normal range. In the early stage of CKD, declining renal function reduces phosphate clearance and klotho production. Compensatory surges in PTH and FGF-23 initially preserve normophosphatemia. However, as GFR progressively declines in advanced CKD, these adaptive responses become inadequate. Diminished urinary excretion, coupled with dysregulated bone metabolism and ongoing dietary intake, ultimately leads to hyperphosphatemia. Hyperphosphatemia is an independent risk factor for all-cause and cardiovascular mortality, particularly in patients with CKD [13].

### 2.2. Dysregulation of Calcium Homeostasis in CKD

Calcium exerts essential functions in a series of physiological processes, including bone mineralization, muscular contraction, neuronal signaling transmission, and coagulation. The vast majority of bodily calcium (approximately 99%) resides in bone tissue as hydroxyapatite, with the remainder distributed in blood and intracellular compartments. Dietary intake is crucial for providing sufficient calcium to maintain healthy body stores. Calcium from dietary sources is mainly absorbed in the small intestine via passive paracellular diffusion and active transcellular transport [14]. The latter, concentrated in the duodenum, involves the participation of transient receptor potential vanilloid 6 (TRPV6) channels, calbindin-D, and PMCA1b ATPase. The kidneys are responsible for the reabsorption of filtered calcium through paracellular and transcellular routes mediated by TRPV5, calbindin-D, Na-Ca exchanger, NCX1, and PMCA1b [15]. Additionally, daily calcium flux occurs between bone and extracellular compartments, regulated by coupled formation and resorption processes.

Systemic calcium balance is orchestrated by endocrine mediators, notably active vitamin D metabolites, PTH, and klotho, which coordinate calcium fluxes across intestinal, renal, and skeletal tissues. 1,25-dihydroxyvitamin D elevates serum calcium through enhanced intestinal absorption, reduced renal excretion, and stimulated bone resorption. By inducing renal 1α-hydroxylase activity, PTH increases the production of active 1,25-dihydroxyvitamin D and thereby augments serum calcium. Klotho also maintains calcium levels via TRPV5-dependent calcium reabsorption in renal tubules [16]. These integrated pathways tightly regulate serum calcium concentrations within a normal range.

In CKD, progressive decline in renal function impairs 1,25-dihydroxyvitamin D synthesis by decreasing renal 1α-hydroxylase activity and elevating FGF23 levels, which directly suppresses 1α-hydroxylation as an inhibitor of 1α-hydroxylase. The resulting 1,25-dihydroxyvitamin D deficiency contributes to hypocalcemia. When combined with hyperphosphatemia, this creates a potent driver for PTH hypersecretion and secondary hyperparathyroidism commonly observed in CKD patients. Long-term exposure to high levels of cyclic PTH results in enhanced bone resorption by osteoclasts, leading to the release of calcium and phosphate into the circulation. Moreover, concurrent use of calcium-based phosphate binders for hyperphosphatemia control further exacerbates calcium accumulation in susceptible patients [17]. These therapeutic interventions, compounded by aberrant skeletal remodeling, collectively disrupt calcium homeostasis in CKD.

### 2.3. Phosphate Imbalance on Vascular Calcification

Vascular calcification is a prevalent and prominent complication in patients with CKD, which is characterized by increased vessel stiffness, leading to elevated systolic blood pressure and adverse cardiovascular events. The development of pathological calcification in CKD patients is primarily driven by dysregulated mineral metabolism, including sustained hyperphosphatemia and hypercalcemia. Like physiological bone formation, vascular calcification is a cell-mediated process in which vascular smooth muscle cells (VSMCs) play an essential role. Elevated extracellular levels of phosphate and calcium have been shown to affect the survival and phenotype of VSMCs, ultimately leading to calcification.

Elevated serum phosphate is recognized as a major risk factor for cardiovascular events in CKD, and the effects of hyperphosphatemia on vascular calcification have been well-studied up to now. Notably, even phosphate levels within the high–normal range (3.5–4.5 mg/dL) independently associate with elevated cardiovascular and all-cause mortality in both CKD cohorts and the general population [18,19]. Thus, phosphate burden, even in the absence of overt hyperphosphatemia, plays a significant role in driving vascular calcification. Potential mechanisms that mediate phosphate-induced VSMC calcification include oxidative stress, osteogenic differentiation, apoptosis, and extracellular matrix (ECM) remodeling.

*Oxidative stress.* Extracellular Pi is mainly transported across the plasma membrane by a ubiquitously distributed solute carrier family, SLC20A, which mainly consists of two members, PiT-1 (SLC20A1) and PiT-2 (SLC20A2). Pi transport via SLC20A is electrogenic and Na^+^-dependent, eliciting inwardly rectifying currents and plasmalemmal depolarization. The voltage-gated calcium channels (VGCCs), highly expressed in VSMCs, are subsequently opened, resulting in Ca^2+^ influx and accumulation of Ca^2+^ in the cytosol, which is essential for Pi-induced oxidative stress and vascular calcification [20]. Silencing of PiT-1 and PiT-2, inhibition of VGCCs by verapamil or nimodipine, and the absence of extracellular Ca^2+^ all prevent oxidative stress in VSMCs [21,22,23]. In addition to the plasmalemmal Pi transporters, mitochondrial Pi transporters deliver Pi from the cytosol to the mitochondria for ATP synthesis, activation of mitochondrial metabolism, and oxidative phosphorylation. These mitochondrial Pi transporters belong to the solute carrier family 25 (SLC25) located in the inner membrane of mitochondria, including phosphate carrier (PiC), dicarboxylate carrier (DIC), ATP-Mg/Pi transporter, and uncoupling protein-2 (UCP2) [20]. Among them, PiC is the predominant route for Pi uptake into the mitochondria, which is driven by the pH gradient. Mutations in SLC25A3, the gene encoding PiC, in humans lead to mitochondrial cardiomyopathy or skeletal myopathy [24,25]. Missense variants in SLC25A24, encoding ATP-Mg/Pi transporter for ATP-Mg and Pi exchange between the cytosol and mitochondria, lead to disrupted transporter dynamics and mitochondrial dysfunction, suggesting that the homeostasis of Pi mitochondrial transport is essential for cellular function [26]. In contrast, excessive uptake of Pi into the mitochondria results in an increase in mitochondrial ROS generation, which serves as the largest ROS pool. The mechanisms include hyperpolarization of the mitochondrial membrane, a decrease in the mitochondrial pH gradient, and acceleration of oxidative phosphorylation [27]. Electrogenic mitochondrial transport of Pi results in mitochondrial hyperpolarization directly, while electroneutral mitochondrial Pi transport is coupled with H^+^ uptake, therefore decreasing the mitochondrial pH gradient and causing mitochondrial hyperpolarization indirectly [28,29]. Moreover, accelerated oxidative phosphorylation causes increased leaking of electrons from the electron transport chain, which react with O_2_ to generate superoxide [30].

Oxidative stress has always been recognized as a contributing factor to vascular calcification in CKD. Serum biomarkers for oxidative stress are higher in patients with stage 5 CKD and vascular calcification than in those without vascular calcification. Serum from patients with stage 5 CKD induces calcium deposition and osteoblastic transition in primary VSMCs by oxidative stress [31]. Natural antioxidants from diets such as procyanidin B2 and quercetin, and from Chinese herbals such as wogonin and celastrol, exhibit anti-vascular calcific effects in VSMCs and CKD animals, while pro-oxidants such as nicotine aggravate vascular calcification via a Ca^2+^ and oxidative stress-dependent manner [32,33,34,35,36]. A systematic review of 77 clinical trials evaluating interventions to attenuate vascular calcification in CKD patients highlighted the efficacy of sodium thiosulfate, which may act as an antioxidant H_2_S donor in vivo [37]. Pi-induced ROS elevation promotes vascular calcification in VSMCs through multiple signaling pathways. The release of mitochondrial ROS into the cytosol of VSMCs under Pi culture contributes to the activation of nuclear factor-κB (NF-κB) signaling, including IKKβ phosphorylation, IκBα degradation, and p65 nuclear translocation, which then acts as a transcription factor to increase the expression of osteogenic genes [38]. In addition, excess Pi activates extracellular signal-regulated kinase (ERK1/2) signaling in VSMCs through the increased levels of mitochondrial ROS, which phosphorylates Runx2 and modulates its transcription activity [23]. Furthermore, mitochondrial ROS activates the phosphatidylinositol 3-kinase (PI3K)/protein kinase B (AKT) pathway by inducing the oxidative inactivation of phosphatase and tensin homolog (PTEN) [39]. Activated AKT then increases PiT-1 trafficking in the plasma membrane, activating mammalian target of rapamycin (mTOR) signaling, which in turn upregulates the expression of PiC, PiT-1, and PiT-2 and promotes oxidative stress via positive feedback [39,40].

*Osteogenic differentiation.* Under high phosphate conditions, VSMCs undergo transdifferentiation from a contractile to an osteoblastic phenotype, characterized by downregulation of contractile markers (α-smooth muscle actin (α-SMA), smooth muscle 22α (SM22α), and calponin) and upregulation of osteogenic gene expressions (bone morphogenetic protein 2 (BMP2), runt-related transcription factor 2 (Runx2), osteopontin, and alkaline phosphatase (ALP)). As a result, osteogenic differentiation of VSMCs induces the release of matrix vesicles containing apatite and calcifying collagen fibrils, which act as nucleation sites for calcification. In a study of human cells, a high phosphate concentration in the medium was found to increase DNA methyltransferase activity and methylation of the SM22α promoter. The increased methylation led to a loss of SM22α, a gain of Runx2, and increased ALP activity, which together facilitated subsequent calcification [41]. BMP2 has been implicated as a pivotal modulator of phosphate-induced VSMC calcification. Kramann et al. reported that the addition of noggin, a BMP-2 inhibitor, effectively blocked the mineralization induced by high-phosphate media and suppressed the expression of bone-related genes [42]. In vivo evidence further supports a key role of osteogenic differentiation in phosphate-induced vascular calcification. Uremic mice fed high-phosphate diets exhibited osteogenic conversion in calcified vessels [43,44]. Similarly, adenine-induced uremic rats exhibited hyperphosphatemia and enhanced osteogenic conversion and calcification [45], which was alleviated by lanthanum carbonate treatment, a phosphate-binding agent that reduces phosphate load [46].

*Apoptosis.* VSMC apoptosis is another essential mechanism by which phosphate induces vascular calcification. Critically, downregulation of growth arrest-specific gene 6 (Gas6) and its receptor Axl is an important factor underlying this process [47]. Apoptotic VSMCs generate apoptotic bodies rich in calcium that serve as nidi for calcium phosphate deposition, directly initiating vascular calcification [48]. Hyperphosphatemia also induces ECM remodeling through multiple pathways, ultimately promoting vascular mineralization. It stimulates the production of matrix metalloproteinases (MMPs) and cysteine proteases that degrade matrix proteins, generate bioactive elastin peptides, and enhance collagen synthesis to create a collagen-enriched ECM [49,50,51]. Upregulation of MMPs has been observed in diabetic CKD arteries and correlates with arterial stiffness [52], and MMP knock-out mice do not develop elastin degeneration and vascular calcification [53]. Concurrently, hyperphosphatemia upregulates enzymes regulating collagen crosslinking and supramolecular organization. In addition, elastin degradation increases the ECM’s affinity for calcium, facilitating hydroxyapatite crystal growth along the elastic lamellae. These elastin fragments also bind elastin laminin receptors on VSMCs, activating transforming growth factor-β (TGF-β) signaling, which promotes VSMC proliferation and upregulates Runx2, ultimately driving osteogenic differentiation [54,55]. Collectively, these mechanisms synergize to remodel the ECM into a mineralization-permissive microenvironment.

### 2.4. Calcium Imbalance on Vascular Calcification

Unlike phosphate, elevated calcium alone does not seem to mediate osteogenic differentiation of VSMCs. It primarily acts as a major nucleator of crystalline hydroxyapatite, initiating the calcification cascade at its earliest stages. Another mechanism whereby elevated extracellular calcium drives VSMC calcification is to promote VSMC matrix vesicle release. Under normal physiological conditions, contractile vascular VSMCs release extracellular vesicles, particularly matrix vesicles, to maintain homeostasis. These matrix vesicles contain calcification inhibitors, including matrix Gla protein (MGP) and circulating fetuin-A. Dialysis patients exhibit diminished fetuin-A concentrations, which are likely to promote calcium-dependent calcification mediated by matrix vesicles. Moreover, VSMCs deplete MGP from matrix vesicles when exposed to elevated calcium, further augmenting their calcification potential. Concurrently, matrix vesicles are loaded with calcium and MMP-2, thereby accelerating elastin degradation and subsequent calcification. Studies in vivo and ex vivo have further demonstrated the significance of increased apoptosis and vesicle deposition in driving extracellular matrix calcification [56].

## 3. Uremic Toxins

Uremic toxins are harmful solutes that accumulate in the body due to impaired renal elimination. Based on their molecular weight and protein-binding characteristics, uremic toxins are typically classified into three categories: free water-soluble low-molecular-weight solutes (<500 Da), middle molecules (>500 Da), and protein-bound uremic toxins (PBUTs). Previous reviews have described endogenously generated uremic toxins, such as β-2 microglobulin, interleukins, and other inflammatory markers. In the present review, we aim to focus on the most toxic dietary and gut-derived compounds.

### 3.1. Indoxyl Sulfate

**Figure 1:**
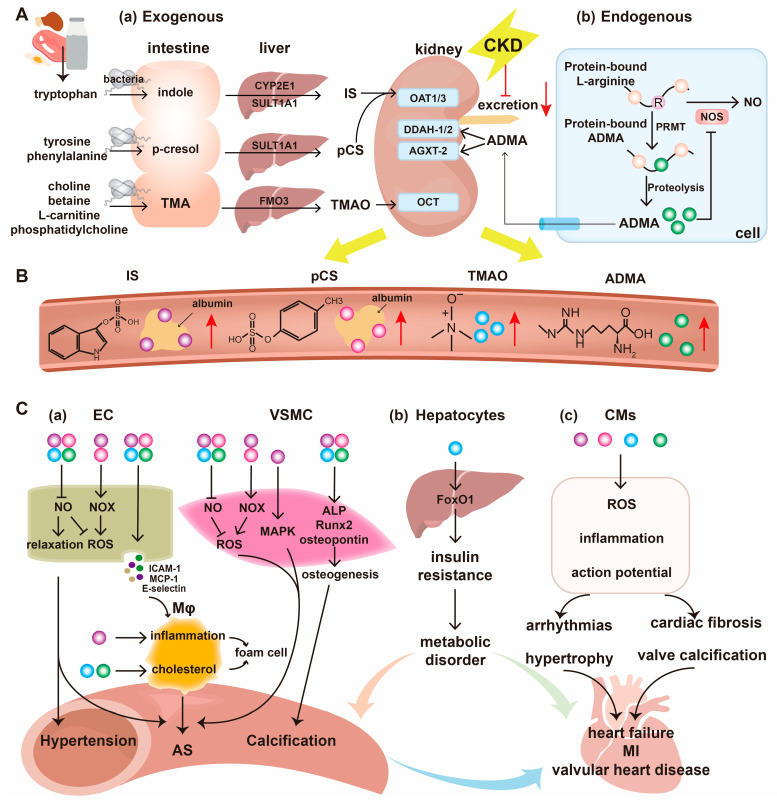
The metabolism and cardiovascular toxicity of uremic toxins. (**A**). The production and excretion of uremic toxins. (**a**) Exogenous gut-derived uremic toxins. Dietary tryptophan is converted into indole by gut bacteria in the intestine, subsequently absorbed, and transported into the liver, where it is catalyzed by cytochrome P450 2E1 (CYP2E1) and sulfotransferase 1A1 (SULT1A1) to generate indoxyl sulfate (IS). P-cresyl sulfate (pCS) originates from dietary tyrosine and phenylalanine, which are metabolized to p-cresol in the intestine by gut bacteria, sulfated by SULT1A1 in the liver, and eventually converted to pCS. Dietary precursors, including phosphatidylcholine, choline, betaine, and L-carnitine, generate trimethylamine (TMA) by bacteria, which is further oxidized by flavin monooxygenase isoform 3 (FMO3) in the liver to form trimethylamine N-oxide (TMAO). The exogenous toxins from foods are excreted in the kidney by organic anion transporters 1/3 (OAT1/3, mediating IS and pCS) and organic cation transporter (OCT, mediating TMAO). (**b**) The endogenous production of uremic toxins. Asymmetric dimethylarginine (ADMA) is produced by protein arginine methyltransferases (PRMT), which methylate the L-arginine in proteins post-translationally and release it after proteolysis. ADMA is metabolized through dimethylarginine-dimethylaminohydrolase-1/2 (DDAH1/2) and alanine glyoxylate aminotransferase 2 (AGXT2) and excreted in the kidney. Under CKD, the excretion of toxins is reduced, leading to increased levels of uremic toxins in the circulation. (**B**). Structural and circulating forms of IS, pCS, TMAO, and ADMA. IS and pCS are bound to albumins in circulation with high affinity. (**C**). The cardiovascular toxicity of uremic toxins. (**a**) Vascular toxicity. In endothelial cells (ECs), the four toxins all decrease NO production and increase the expression of inflammatory cytokines and selectins, resulting in reactive oxygen species (ROS) production, decreased relaxation, and inflammation. In addition, IS and pCS upregulate NADPH oxidase (NOX) expression to increase ROS. In vascular smooth muscle cells (VSMCs), the four toxins similarly increase ROS production as in ECs. Moreover, the four toxins upregulate the expression of osteogenic runt-related transcription factor 2 (Runx2), alkaline phosphatase (ALP), and osteopontin, promoting vascular calcification. In macrophages (Mφ), IS increases the inflammation level, while TMAO and ADMA increase cholesterol accumulation, both promoting the formation of foam cells. The pro-oxidant, pro-inflammatory, and osteogenic effects of the four toxins contribute to the development of hypertension, atherosclerosis, and calcification. (**b**) Metabolic toxicity. Liver-synthesized TMAO upregulates the expression of forkhead box protein O1 (FoxO1) and aggravates insulin resistance and metabolic disorder. (**c**) Cardiac toxicity. The four toxins increase the ROS production and inflammation in cardiomyocytes (CMs), prolong the action potential duration, and induce early afterdepolarization, all of which result in cardiac arrhythmias, hypertrophy, fibrosis, and valve calcification, leading to heart failure, myocardial infarction (MI), and valvular heart disease. The vascular, metabolic, and cardiac toxicities aggravate each other via positive feedback, promoting the development of cardiovascular diseases. Red arrows: increased levels of uremic toxins. Purple circles: IS. Pink circles: pCS. Blue circles: TMAO. Green circles: ADMA.

Indoxyl sulfate (IS) originates from the bacterial metabolism of tryptophan [57]. Dietary tryptophan is metabolized through distinct pathways. The majority of tryptophan can be metabolized through the kynurenine metabolic pathway, mediated by tryptophan 2,3-dioxygenase (TDO) and indoleamine 2,3-dioxygenase (IDO). A minor amount of tryptophan undergoes metabolism through the serotonin pathway, which leads to melatonin, and the indolic pathway, which leads to indole [6]. The generation of indole is mediated by bacterial tryptophanase and is absorbed into the portal circulation to be subsequently transported to the liver [58] (Figure 1). Within the liver, indole undergoes hydroxylation catalyzed by cytochrome P450 2E1 (CYP2E1) to form 3-hydroxy indole (indoxyl), which is the major isoform for the microsomal oxidation of indole to indoxyl [59]. Then the indoxyl is transformed into IS by sulfotransferase 1A1 (SULT1A1) [60]. In addition to IS, indoxyl also undergoes hepatic glucuronidation to yield indoxyl glucuronide and is metabolized by aryl acetate-transferase to generate indoxyl acetate [6,61]. IS undergoes substantial renal excretion into the urine, primarily via the basolateral organic anion transporter 1 (OAT1) and OAT3 in the proximal tubules [62]. In the systemic circulation, IS exhibits a high binding affinity to albumin (Table 1), rendering its removal by dialysis inefficient [63].

#### 3.1.1. Mechanisms for the Progression of CKD

In kidney failure, the accumulation of IS results from increased production in the gut due to gut dysbiosis, alterations in enzyme activity, and ineffective renal excretion [6]. This elevation in IS concentration further contributes to the progression of CKD and increased risk of cardiovascular damage. After tubular intake, IS could activate the aryl hydrocarbon receptor (AhR), directly inducing apoptosis of renal tubular epithelial cells and renal injury [64]. The binding of IS to AhR also enhances the production of ROS while simultaneously reducing the activity of superoxide dismutase and glutathione within cells. This leads to increased oxidative stress and diminished antioxidant capacity, thereby exacerbating tubular epithelial damage. The impaired tubular cells subsequently secrete excessive transforming growth factor-β1 and induce kidney fibrosis. Epithelial–mesenchymal transition (EMT) is a crucial pathological process of renal tubulointerstitial fibrosis. IS has been found to induce EMT of tubular epithelial cells in vivo and in vitro via activation of MAPK [65], PI3K/Akt signaling [66], and the renin–angiotensin–aldosterone system [67], further deteriorating kidney function. In human proximal tubular cells (HK2), IS stimulation downregulates klotho expression and inhibits the Akt/Nrf2 axis, inducing mitochondrial dysfunction, ROS accumulation, and, ultimately, apoptosis [68]. Moreover, recent evidence indicates that IS promotes renal ferroptosis in the progression of CKD by suppressing the expression of selenium transporter SEPP1 and selenoprotein GPX4 [69].

#### 3.1.2. Mechanisms for the Progression of CVD

Emerging evidence suggests that IS may contribute to a wide spectrum of cardiovascular diseases in CKD patients [70]. In a prospective study of 147 patients with stage 1–5 CKD, elevated serum IS levels were independently associated with major adverse cardiovascular events after adjusting for GFR and nutritional status [71]. Moreover, IS is involved in the pathogenesis of atherosclerosis by negatively correlating with high-density lipoprotein cholesterol [72,73].

IS has shown cardiovascular toxicity by inducing endothelial dysfunction, oxidative stress, and inflammation [74,75,76]. Endothelial dysfunction is widely recognized as an early marker for atherosclerosis. In CKD, oxidative stress and nitric oxide (NO) deficiency are key contributors to the development of endothelial dysfunction. From blood samples obtained from 110 patients with stage 3–5 CKD, it could be concluded that IS levels are inversely associated with vascular reactivity index values and have a modulating role in endothelial function [77]. IS inhibits NO production and cell viability, induces ROS generation, and upregulates NADPH oxidase 4 (NOX4) expression in vascular endothelial cells and VSMCs [78,79]. Moreover, IS accumulated in CKD can upregulate miR-92a, a microRNA induced by oxidative stress in endothelial cells and involved in angiogenesis and atherosclerosis [80]. Furthermore, IS participates in the pathophysiology of atherosclerosis by upregulating the expression of monocyte chemotactic protein 1 (MCP1), intercellular adhesion molecule-1 (ICAM-1), and E-selectin, inducing leukocyte–endothelial interactions and promoting inflammation [81,82]. Nakano et al. reported that IS triggers Notch signaling and promotes proinflammatory macrophage activation in atherosclerosis [83]. Additionally, IS activates the MAPK pathway and directly stimulates VSMC proliferation, a key event in the progression of atherosclerosis [84].

Another common vascular complication associated with CKD is vascular calcification. Barreto et al. previously demonstrated that serum IS levels exhibit an inverse relationship with renal function and a direct relationship with aortic calcification and pulse wave velocity in patients with different stages of CKD [85]. Both in vivo and in vitro studies have shown that IS induces aortic calcification with increased expression of osteoblast-specific proteins, including Runx2, ALP, and osteopontin [78,86]. This osteogenic effect of IS is partially mediated through activation of the PI3K/Akt/NF-κB signaling pathway and suppression of klotho expression [87,88]. Furthermore, IS accelerates VSMC senescence and activates inflammatory pathways, which together facilitate vascular calcification [89,90].

Dietary tryptophan-derived IS also contributes to the progression of peripheral artery disease (PAD). In a prospective cohort of PAD patients, plasma IS levels significantly increased the risk of future adverse limb events. Similarly, IS suppressed endothelial progenitor cell-mediated neovascularization in ischemic hindlimbs [91]. Regarding the mechanism, IS downregulates the Wnt/β-catenin axis pathway, leading to endothelial cell dysfunction and impaired angiogenesis [92].

IS is proposed to have a detrimental effect not only on the vasculature but also on cardiac cells. IS has been shown to exert pro-fibrotic, pro-hypertrophic, and pro-inflammatory effects via activation of the MAPK and NF-κB pathways [93]. Moreover, IS could prolong the action potential duration and induce early afterdepolarization, which is known to be a trigger mechanism of lethal ventricular arrhythmias [94]. In hypertensive rats, IS aggravated cardiac fibrosis and cardiomyocyte hypertrophy by enhancing oxidative stress while suppressing antioxidant defenses [95,96]. Clinically, cross-sectional studies have revealed significant negative correlations between serum concentrations (total and free) of IS and peak cardiac power, alongside positive associations with subclinical cardiac dysfunction [97]. Moreover, elevated plasma IS levels are associated with an increased risk of left ventricular diastolic dysfunction and incident heart failure in hemodialysis patients [98,99].

### 3.2. P-Cresyl Sulfate

Similar to IS, p-cresyl sulfate (pCS) is also an amino acid metabolite derived from gut bacteria. Briefly, tyrosine and phenylalanine are metabolized to 4-hydroxyphenylacetic acid, which is subsequently decarboxylated to p-cresol. P-cresol is absorbed by the liver and then sulfated, primarily by SULT1A1, to form pCS (Figure 1) [100]. The resulting pCS is predominantly secreted via OATs located on the basolateral membrane of renal tubules and ultimately excreted in the urine [101]. In patients with CKD, impaired renal function leads to a significant accumulation of pCS in the systemic circulation. Due to its high binding affinity for albumin (Table 1), pCS is poorly cleared by conventional dialysis modalities [102]. This substantial accumulation of pCS induces further renal damage and contributes to the development of cardiovascular complications.

#### 3.2.1. Mechanisms for the Progression of CKD

Similar to the nephrotoxicity of IS, pCS enhances the activity of NADPH oxidase and increases the production of ROS, leading to renal tubular cell toxicity. As a result, the expression of TGF-β1, TIMP-1, and Pro-α1 (I) collagen is upregulated, which induces renal fibrosis. Moreover, pCS compromises cellular antioxidant defenses by decreasing glutathione levels, leading to increased susceptibility to oxidative stress [103]. In vivo evidence also shows that administration of pCS to nephrectomized rats causes significant renal damage by increasing oxidative stress [104]. Klotho is a transmembrane protein, and some studies have suggested its protective role in the kidneys [105]. pCS has been shown to increase CpG hypermethylation of the klotho gene and result in reduced klotho expression, promoting tubulointerstitial fibrosis formation and CKD progression [106]. Furthermore, caspase-3-mediated apoptosis and acceleration of the cell cycle are involved in pCS-induced toxicity to renal tubular cells [107].

#### 3.2.2. Mechanisms for the Progression of CVD

In patients with CKD, serum-free pCS levels have been reported to predict the risk of all-cause and cardiovascular mortality beyond traditional and uremia-related risk factors [108,109]. Multivariate Cox regression analysis was used in these prospective studies for adjustment of age, gender, blood pressure, diabetes, albumin, eGFR, and other possible confounders and validated in different cohorts [110]. Additionally, serum total pCS levels are significantly related to cardiovascular events, which are higher in coronary artery disease and are correlated with the severity of the disease [111,112].

pCS contributes to atherosclerosis through multiple mechanisms, including the induction of oxidative stress, promotion of inflammation, impairment of endothelial function, and stimulation of VSMC proliferation. pCS induces oxidative stress by enhancing the production of ROS and activating leucocyte free radical production [111,113,114]. In vitro studies have shown that pCS may play a role in endothelial dysfunction through enhancing NADPH oxidase expression and ROS production [111], as well as inducing shedding of endothelial microparticles [115]. Furthermore, pCS adversely affects endothelial progenitor cells (EPCs). Notably, the number of EPCs inversely correlates with markers of vascular injury, including pulse wave velocity and endothelial microparticle levels [116]. pCS also promotes VSMC proliferation and migration while disrupting the balance between matrix metalloproteinases and tissue inhibitor of metalloproteinases within atherosclerotic plaques, thereby accelerating plaque progression [117].

In a cohort study by Liabeuf et al., total and free pCS presented an inverse relationship with renal function and were significantly associated with vascular calcification [118]. In vitro studies have also demonstrated that pCS promotes vascular calcification, as evidenced by elevated mRNA levels of ALP, Runx2, and osteopontin [111]. Mechanistically, pCS-induced ROS activates the phosphorylation of ERK, JNK, and p38. This, in turn, enhances NF-κB-dependent transcription of Runx2 and ALP [119]. In addition, pCS exerts cardiac toxicity by inducing NADPH oxidase activity and ROS production, thereby facilitating cardiac apoptosis and resulting in diastolic dysfunction [120].

### 3.3. Trimethylamine N-Oxide

Trimethylamine N-oxide (TMAO) is a gut-derived amine oxide that has garnered significant attention due to its association with various health conditions, particularly CKD and CVD. TMAO is produced from dietary precursors such as phosphatidylcholine, choline, betaine, and L-carnitine, which are abundant in foods such as red meat, fish, and eggs. Upon ingestion, these compounds are metabolized by gut bacteria into trimethylamine (TMA), which is rapidly absorbed into the circulation and oxidized in the liver by flavin monooxygenase isoform 3 (FMO3) to form TMAO [121] (Figure 1). TMAO elimination occurs via active secretion mechanisms through different transporters, among which organic cation transporters (OCTs) represent the principal mediators [122]. The levels of TMAO can be significantly elevated in individuals with CKD, which is likely attributable to its potentially increased production and reduced renal filtration [6].

#### 3.3.1. Mechanisms for the Progression of CKD

TMAO has been identified as a potential biomarker for predicting the progression of CKD and adverse events in CKD patients. Plasma TMAO levels are markedly higher in CKD subjects than in non-CKD subjects, and higher levels of TMAO are independently associated with a 2.8-fold increase in mortality risk [123]. In a large prospective study enrolling 10,564 participants without impairment in eGFR at baseline, higher TMAO levels were associated with a higher risk of incident CKD and greater annualized eGFR decline in a median follow-up of 9.4 years, which was consistent across different racial or ethnic groups. In addition, the association between TMAO and eGFR decline was similar to or greater than the well-established CKD risk factors [124]. Inhibiting, inducing, or adding TMAO in rodents with CKD or in a cellular model of CKD has revealed that TMAO promotes renal dysfunction by aggravating renal fibrosis, inflammation, and oxidative stress. In human renal fibroblasts, TMAO increases the total collagen production via PERK/AKT/mTOR signaling; promotes the proliferation of fibroblasts through nucleotide-binding domain, leucine-rich-containing family, pyrin domain-containing-3 (NLRP3), and caspase-1; and enhances TNF-α-mediated inflammation and fibrosis [125,126]. In mice or rats with diet-induced obesity, diabetic kidney disease, or adenine-induced CKD, TMAO aggravates renal fibrosis and dysfunction by increasing oxidative stress, NLRP3-mediated inflammation, and renal tubular ferroptosis [127,128,129]. Different inhibitors of TMAO production, including 3,3-dimethyl-1-butanol (DMB) and iodomethylcholine (IMC), which inhibit TMA lyase, traditional Chinese medicine downregulating the gut microbiota related to TMAO, and antibiotic-induced microbiota depletion, alleviate renal fibrosis and improve renal function in the progression of CKD [127,129,130,131].

#### 3.3.2. Mechanisms for the Progression of CVD

A high TMAO concentration has been linked to a range of cardiovascular events and cardiac death in hemodialysis patients [132]. Serum TMAO concentrations substantially increase with decrements in kidney function, and increased TMAO concentrations correlate with coronary atherosclerosis burden and risk of ischemic cardiovascular events [133,134]. TMAO reduces NO production and increases vascular oxidative stress and inflammation, contributing to CKD-associated endothelial dysfunction and cardiovascular disease [135,136]. As a result, TMAO becomes a causative factor in the development of PAD by impairing endothelium-derived hyperpolarizing factor-type relaxation [137].

Experimental data suggest that TMAO may directly promote atherogenesis through mechanisms involving the dysregulation of lipid metabolism and macrophage function, as well as by inducing vascular inflammation and platelet activation [138,139]. Dietary supplementation with choline or TMAO elevates plasma TMAO levels and exacerbates aortic atherosclerosis in apolipoprotein E knockout mice, accompanied by increased macrophage content compared to wild-type controls [140]. Elevated TMAO levels also reduce reverse cholesterol transport and activate the CD36/MAPK/JNK and NF-κB pathways, which induce the formation of foam cells and the expression of inflammatory factors [141,142]. Furthermore, enhanced platelet activation and adhesion have been observed following TMAO injection, indicating its role in thrombus formation [143].

TMAO is also implicated in the pathogenesis of vascular calcification. Plasma levels of TMAO have been found to be higher in hemodialysis patients with a high abdominal aortic calcification score compared to those with a low score [144]. TMAO upregulates the expression of osteoblast-specific proteins and induces VSMC and arterial ring calcification. In vivo, exogenous administration of TMAO in mice aggravates subtotal nephrectomy-induced vascular calcification. Activation of NLRP3 inflammasome and NF-κB signals by TMAO is involved in its osteogenic effects [145].

Studies have also suggested that TMAO may elicit detrimental effects on cardiomyocytes. High TMAO levels have been observed in patients with heart failure (HF), and this elevation is associated with an increased risk of adverse outcomes in both acute and chronic HF [146,147,148]. Dietary choline-mediated elevation in circulating TMAO levels exacerbates adverse cardiac remodeling, which can be attenuated after dietary modification to reduce the choline/TMAO content in a HF model [149]. Moreover, TMAO-induced oxidative stress injury is involved in cardiac diastolic dysfunction of HF with preserved ejection fraction [150]. A recent prospective study revealed that TMAO is strongly associated with left ventricular diastolic dysfunction and adverse remodeling following ST-elevation myocardial infarction and may help identify such patients for early treatment [151].

Several experimental studies have demonstrated that TMAO promotes cardiac hypertrophy [152] and fibrosis via the TGF-β1 and NF-κB signaling pathways, directly impairing cardiomyocyte contractility and cardiac function [153,154,155]. Moreover, TMAO has been identified as a prognostic marker in valvular heart diseases because of its facilitation of aortic valve fibrosis and inflammation [156,157].

Additionally, recent findings have revealed that TMAO promotes metabolic dysfunction by directly binding and activating protein kinase R-like endoplasmic reticulum kinase, a crucial sensor of intracellular stress. This activation upregulates forkhead box protein O1, driving insulin resistance and metabolic disturbances. This newly identified pathway connects TMAO to systemic metabolic dysfunction, providing critical insights into its role in cardiometabolic disease pathogenesis [158].

### 3.4. Asymmetric Dimethylarginine

Asymmetric dimethylarginine (ADMA) is a toxic non-proteinogenic amino acid. As an endogenous competitive inhibitor of nitric oxide synthase (NOS), ADMA can inhibit NOS activity and reduce the synthesis of NO [159]. NOS uses L-arginine as a substrate to produce NO and L-citrulline. ADMA is produced through post-translational methylation of arginine in proteins catalyzed by a family of protein arginine methyltransferases (PRMTs), mainly PRMT1 (Figure 1). A healthy adult generates approximately 60 mg of ADMA daily, with roughly 20% being excreted in urine through renal filtration in the form of prototype [160]. The majority of ADMA is degraded by dimethylarginine-dimethylaminohydrolase-1 (DDAH1) and -2 (DDAH2), which hydrolyze ADMA into dimethylamine (DMA) and L-citrulline. The two isoforms have similar activity but different tissue distribution and are highly expressed in the kidney. The third and secondary route for ADMA elimination is to be metabolized by alanine glyoxylate aminotransferase 2 (AGXT2), a mitochondrial aminotransferase that is highly expressed in the kidneys [161,162]. With the central role of the kidneys in ADMA excretion and metabolism by DDAH and AGXT2, renal impairment on the urinary excretion or the expression and activity of DDAHs and AGXT2 may affect the plasma ADMA levels [163].

#### 3.4.1. Mechanisms for the Progression of CKD

Numerous clinical studies have demonstrated that circulating ADMA levels are elevated in patients with CKD as renal function declines. In a cohort composed of stage 3–4 CKD patients, the mean ADMA concentrations were significantly higher than in the healthy subset. Elevated ADMA was an individual predictor of high risk for adverse kidney outcomes, including increased morbidity, mortality, and graft dysfunction in kidney transplant recipients [164,165]. Opposite findings were observed in childhood CKD patients, whose eGFR was poorly related to the plasma ADMA levels [166]. These discrepancies could be related to the fact that the decrease in renal excretory function is not necessarily paralleled by the reduction in renal DDAH and AGXT2 activity. Said et al. determined the plasma levels and urinary excretion of ADMA in kidney donors before and after donation. As the measured GFR (mGFR) of the donors dropped significantly after donation, the urinary ADMA excretion also decreased while plasma ADMA increased only slightly, probably due to an enhanced metabolism of ADMA as a compensatory response supported by the elevated plasma levels of citrulline [167]. In contrast, in cohorts enrolling unselected humans, the plasma levels of ADMA were correlated with single-nucleotide polymorphisms (SNPs) in DDAH1 and DDAH2, rather than PRMT1 or AGXT2 [168]. DDAH1-transgenic mice showed a 2-fold reduction in plasma ADMA, while DDAH1-deficient mice exhibited increased levels of plasma ADMA [169,170]. These findings suggest that the plasma ADMA is under complicated dynamic control and the metabolic route is the major factor affecting its level, not only in healthy humans but also in CKD patients. In clinical practice, urinary ADMA excretion could be evaluated by the urinary concentration of ADMA corrected by the urinary concentration of creatinine measured in the same spot urine samples. As the metabolites of endogenous ADMA contribute up to 90% of urinary DMA, the urinary creatinine-corrected excretion rate of DMA could be used to evaluate the whole-body ADMA metabolism [171].

However, the role of ADMA in the pathogenesis of CKD remains controversial. As the endogenous inhibitor of NOS, ADMA compromises the integrity of the glomerular filtration barrier at concentrations found in the circulation of patients with CKD by inhibiting the NO-induced 3’,5’-cyclic guanosine monophosphate (cGMP) production [172]. In addition, ADMA induces the assembly of stress fibers and NF-κB activation, increases the expression of TGF-β1 in an actin cytoskeleton-dependent manner in renal glomerular endothelial cells, increases apoptosis in mesangial cells via activation of endoplasmic reticulum stress, and promotes fibrotic alterations in renal fibroblasts and mesangial cells involving ROS-induced ERK activation [173,174,175]. These findings demonstrate the pro-fibrotic and harmful effects of ADMA on glomerular structure and function in CKD. Administration of ADMA for 8 weeks in uninephrectomized mice increased renal oxidative stress and fibrosis but reduced peritubular capillaries, thus resulting in deteriorative renal dysfunction [176]. Bupropion, a dopamine–norepinephrine reuptake inhibitor that decreases circulation ADMA levels, ameliorates renal interstitial lesions and fibrosis by upregulating DDAH1 [177]. Overexpression of DDAH1 decreased plasma levels of ADMA and alleviated the deterioration of renal dysfunction in rats that underwent 5/6 nephrectomy by inhibiting peritubular capillary loss and tubulointerstitial fibrosis [178]. Meanwhile, DDAH1 global knockout aggravated renal dysfunction, interstitial fibrosis, and epithelial-mesenchymal transition (EMT) of tubular epithelial cells in aged or diabetic mice via decreased NO and increased ROS levels [179]. In contrast, other studies have shown opposite findings regarding the protective role of ADMA in renal diseases. Tomlinson et al. revealed that proximal tubule-specific DDAH1 knockout resulted in the accumulation of ADMA in renal tubules and lower NO concentrations, without alterations in plasma ADMA levels. These mice were protected from reduced kidney tissue mass, collagen deposition, and profibrotic cytokine expression in two independent renal injury models: folate nephropathy and unilateral ureteric obstruction (UUO) [180]. In UUO and Adriamycin-induced renal injuries, the expression of renal PRMT1 increased, accompanied by ADMA accumulation [181,182]. PRMT1 inhibitor reduced ADMA production, but increased renal fibrosis in UUO-treated mice [182]. Although PRMT1 plays the major role in asymmetric demethylation of proteins, accounting for 70–80% of ADMA generation, other PRMTs, including PRMT3, are also able to asymmetrically dimethylate proteins, which were increased in UUO-treated mice as well [183]. Consistent with PRMT1 inhibition, PRMT3 deficiency inhibits ADMA production and increases renal fibrosis, blocked by intra-renal injection of ADMA [183]. The discrepancies between studies are probably related to whether ADMA is affected systemically or only within the kidney. When ADMA is elevated in the circulation by exogenous administration or genetic disruption of metabolic enzymes, the increased ADMA exerts a detrimental effect on the kidneys. However, when ADMA accumulation is restricted to the kidneys by conditional genetic disruption or intrarenal administration, the increased ADMA shows a beneficial effect on renal fibrosis. Future applications of ADMA-lowering strategies, including decreasing plasma ADMA directly, inhibiting DDAH activity, or antagonizing PRMTs, should be conducted cautiously for patients with CKD, with concern for the side effects of conditional interventions.

#### 3.4.2. Mechanisms for the Progression of CVD

Elevation of ADMA is associated with increased risk for all-cause mortality and various cardiovascular diseases, such as coronary artery disease, hypertension, and vascular calcification [184,185,186,187]. As previously discussed, the most well-known effect of ADMA is the inhibition of NO production via competitive inhibition of eNOS. Deleting the *Ddah1* gene in mice and using DDAH1-specific inhibitors, plasma ADMA levels increased and NO production decreased. Functional tests showed endothelial dysfunction in the gene-disrupted mice, evidenced by increased contraction in response to phenylephrine and reduced relaxation in response to acetylcholine [170]. NO participates in regulating vascular dilation, reducing excessive proliferation of VSMCs, and inhibiting platelet aggregation and inflammatory cell adhesion and plays crucial endothelial protective roles [188]. Endothelial dysfunction caused by the imbalance in the production and degradation of NO is an important early feature of cardiovascular diseases [189]. ADMA has been shown to inhibit NO synthesis both in vivo and in vitro [190], and vascular endothelial-specific DDAH-knockout mice exhibit increased ADMA levels and reduced NO production and vessel relaxation in isolated aortic rings [191]. Additionally, ADMA is involved in the development of hypertension by regulating the NO–ROS balance [192]. A recent study has reported that ADMA is a novel independent risk factor for coronary artery calcification in patients with CKD [193]. ADMA is not only associated with vascular endothelial dysfunction but also with vascular calcification, both of which concurrently contribute to atherosclerosis progression [194].

Studies have shown that ADMA causes arteriosclerotic coronary lesions through mechanisms other than simple inhibition of endothelial NO synthesis. Direct activation of the renin–angiotensin system and increased oxidative stress are also involved in the long-term vascular effects of ADMA in vivo [195,196]. Furthermore, accumulation of ADMA upregulates the expression of scavenger receptor LOX-1 and increases oxLDL uptake, thus contributing to lipid accumulation and foam cell formation [197]. Another in vitro study showed that the ADMA/DDAH2 axis plays a crucial role in regulating cholesterol metabolism in macrophages. ADMA impaired cholesterol efflux in response to oxLDL by downregulating the expression of ATP-binding cassette transporter A1 (ABCA1) and ABCG1, and the activation of the NADPH oxidase/ROS pathway was involved in this inhibitory effect [198].

## 4. Therapeutic Strategies for Specific Uremic Toxins

The management of CKD is multifaceted, including improving quality of life, alleviating the progression of renal impairment, and preserving the residual renal function, to prepare for a kidney transplant [199]. In clinical practice, while improving renal function and kidney transplantation remain cornerstone strategies, uremic toxin elimination also contributes to the alleviation of CKD. First, reducing the symptom burden to improve quality of life is as important to many CKD patients as extended survival. Theoretically, improving kidney function can reduce plasma toxins, and direct mitigation of uremic toxins may relieve toxin-associated symptoms, such as pruritus, fatigue, and neurocognitive dysfunction [200,201,202]. Second, uremic toxin clearance therapies could augment the effects of reno-protective drugs [203]. Third, the time on the transplantation waiting list takes several years, and cardiovascular events may cause early death before surgery. Thus, toxin reduction preserves cardiovascular homeostasis, critical for transplant candidacy and outcomes. Here, we discuss current therapies for uremic toxin elimination to increase understanding and identify shortcomings.

For water-soluble uremic toxins like TMAO, dialysis would be an appropriate method to decrease their circulating levels. For PBUTs with a high protein binding affinity like IS and pCS, the toxins mainly exist in bound form, which is difficult to remove using conventional dialysis treatment. In recent years, the advancement of dialysis membrane materials has significantly enhanced the efficacy of toxin removal in patients with CKD. Newly developed synthetic membranes, such as polyethersulfone and polyacrylonitrile, exhibit superior permeability and biocompatibility. These new materials not only improve the clearance of uremic toxins, such as IS and pCS, but also reduce the inflammatory response associated with dialysis treatments. Studies have demonstrated that the use of high-flux membranes can significantly enhance the removal of these toxins, leading to improved clinical outcomes, including reduced cardiovascular morbidity and mortality in CKD patients [204]. Furthermore, the incorporation of biocompatible coatings on dialysis membranes is being explored to further minimize the activation of the complement system and reduce the risk of dialysis-related complications.

Gut dysbiosis has been implicated in the pathogenesis of CKD and its associated cardiovascular complications. For gut-derived uremic toxins, the use of oral adsorbents has shown promise in binding uremic toxins within the gastrointestinal tract, thereby preventing their absorption into the bloodstream. AST-120, an artificially synthesized carbon-based adsorbent, is one of the most widely studied oral agents at present. Animal studies have demonstrated that AST-120 can effectively lower serum IS and pCS levels in CKD rats while delaying renal function deterioration [205,206,207]. A meta-analysis revealed that, compared to the placebo, non-dialysis CKD patients treated with AST-120 exhibited a mean reduction of 7.73 mg/L in serum IS levels [208]. Another case–control study indicated that chronic dialysis patients receiving AST-120 treatment experienced significant reductions in serum total pCS and IS levels after three months, with average decrease rates of 32.7% and 21.9% respectively [209].

In addition to enhancing the clearance of gut-derived uremic toxins, modulating the intestinal bacteria-mediated production of uremic toxins is another important therapeutic strategy. The intestinal flora in CKD patients undergoes significant alterations, and an increase in intestinal pH renders the microenvironment alkaline. This shift promotes the overgrowth of aerobic bacteria, which are known to produce indole and p-cresol. Supplementation with prebiotics or probiotics may decrease the intestinal pH, thereby inhibiting the excessive proliferation of aerobic bacteria, enhancing the abundance of saccharolytic bacteria, reducing proteolytic bacteria, and competitively suppressing the growth of bacteria responsible for the production of protein-bound uremic toxin precursors [210]. Common prebiotics include fructooligosaccharides, galactooligosaccharides, resistant starch, and polyphenolic compounds. Representative probiotics encompass Lactobacillus and Bifidobacterium. Synbiotics represent a combination formulation comprising both prebiotics and probiotics. A meta-analysis revealed that the average serum IS level in CKD patients was decreased by 4.66 mg/L, and the average pCS level was decreased by 3.31 mg/L following the use of prebiotics. After the administration of synbiotics, the average serum IS and pCS levels were decreased similarly [208]. Another meta-analysis showed that probiotics could improve the gastrointestinal disorders of dialysis patients and reduce the levels of PBUTs [211]. Overall, the administration of prebiotics, probiotics, and synbiotics has been reported to yield positive effects in reducing the production of IS and pCS in healthy subjects, CKD patients, and hemodialysis patients [210,212,213].

Dietary modification is also indispensable for reducing uremic toxin production by gut bacteria in CKD patients. As IS and pCS originate from the gut bacterial metabolism of tryptophan and tyrosine/phenylalanine, dietary limitation of these precursor amino acids effectively suppresses production of these uremic toxins. Clinical evidence demonstrates that healthy individuals on protein-restricted regimens exhibited significantly lower plasma concentrations and 24 h urinary excretion of IS compared to high-protein comparator groups [214]. Compared to the conventional diet, CKD patients who received a very low protein diet for 6 months had 69% and 58% reductions in serum total IS and pCS levels, respectively [215], indicating that restricting protein intake in the diet can reduce the production of IS and pCS. Notably, dialysis patients are prone to protein-energy wasting and suffer from malnutrition. Therefore, regular monitoring of nutritional indicators is necessary when CKD patients are subjected to a protein-restricted diet. Aromatic amino acids (AAAs) serve as essential precursors for neurotransmitters, skeletal health, and metabolic regulation in humans [216]. Therefore, whether low-AAA diets compromise these vital physiological processes is a critical consideration for CKD patients. First, analysis from the Cardiovascular Health Study enrolling 5178 persons aged ≥ 65 years revealed no significant association of dietary AAA intake with hip fractures, areal bone mineral density of the hip, or body composition. Among those with a hip fracture, the serum levels of phenylalanine or tyrosine showed no significant difference compared to those without hip fractures [217,218]. Second, the most important concern regarding low-AAA diets is the inhibition of neurotransmitters and the effects on neurobehavior. Complete AAA deprivation (0% AAA in diets) reduces the levels of serotonin, 5-hydroxyindoleacetic acid, dopamine, and norepinephrine in some regions of the brain, concomitant with alterations in food intake and behavior [219]. However, complete and long-term AAA deprivation in diets is impractical in clinical trials. Acute AAA depletion in healthy volunteers decreased serum tyrosine to 80%, at which level no mood effects were observed [220]. In a clinical trial by Hayware et al., healthy volunteers were given a tryptophan-free or a control mixture, with 0.007% and 0.023% tryptophan, respectively, leading to a 50% reduction of serum tryptophan levels, and no alterations in mood were observed [221]. A low-AAA diet of a similar formula (14% proteins, 0.007% AAA) was given to mice with adenine-induced CKD, which reduced proteinuria, renal fibrosis, and inflammation, compared to those fed a normoproteic diet (14% proteins, 0.019% AAA). Although low protein diets with normal levels of AAA (5% proteins, 0.019% AAA) and low-AAA diets both alleviated renal impairment in CKD, plasma free pCS and IS were only slightly decreased in mice fed low protein diets with normal levels of AAA but significantly decreased in mice fed low-AAA diets [222]. The body weight, metabolic parameters, and hemodynamics were not influenced by AAA restriction, suggesting long-term safety. In CKD patients, monitored reduction (not depletion) of plasma AAAs through tailored diets achieves uremic toxin-lowering benefits without crossing thresholds for physiological impairment.

Pharmacologically increasing DDAH activity is the foremost therapeutic strategy being explored for ADMA reduction. Experimental studies have shown that upregulation of ADMA expression and activity reduces circulating ADMA and yields multiple vascular benefits [159]. However, the pharmacological development of specific DDAH activators confronts persistent research challenges. Since L-arginine serves as the substrate for NOS in the production of NO, supplementation with L-arginine and L-citrulline (the precursor of L-arginine) is another feasible therapeutic strategy for reducing ADMA levels and enhancing NO bioavailability [223,224,225].

## 5. Conclusions and Future Directions

The intricate interplay between CKD and CVD is underscored by a complex web of metabolic disturbances that establishes a vicious cycle detrimental to both organ systems. As we explored throughout this review, traditional mineral imbalances, particularly in calcium and phosphate, alongside the accumulation of uremic toxins, serve as critical mediators in this pathological relationship. The impact of disrupted calcium phosphate metabolism on vascular calcification exemplifies how CKD can directly compromise cardiovascular health, while the multifaceted effects of uremic toxins introduce a further layer of complexity through mechanisms such as oxidative stress, inflammation, and fibrosis. Despite the progress made in elucidating these mechanisms, significant gaps remain in our understanding of the pathological roles of many uremic toxins. Delineating the specific pathways through which these toxins exert their effects is essential for advancing our knowledge and informing therapeutic strategies.

Monitoring the levels of uremic toxins in the blood is an important means to understand the degree of toxin accumulation and the adequacy of dialysis in CKD patients. Although current follow-up with the Ca × P product has offered predictive value in the CKD progression, it may be insufficient in predicting cardiovascular events. However, adding free p-CS and IS to the Ca × P product enhances its value in predicting all-cause and cardiovascular mortality in hemodialysis patients [108]. In addition, with the development of techniques, toxin measurement has become feasible. A novel detection method, combining a plasma separation/absorption pad and nanozyme, could be applied for direct quantitative detection of TMAO with a wide quantification range using only a drop of blood [226]. Liquid chromatography tandem mass spectrometry methods enable the simultaneous quantification of seven uremic toxins from a small volume of serum (50 µL) within minutes [227]. Meanwhile, most toxins are routinely detected in clinical practice currently. For some highly toxic PBUTs that are closely related to the prognosis of patients, such as IS and pCS, there is an urgent need to develop a method suitable for routine detection and real-time monitoring. Furthermore, although an increasing number of toxins are being identified and characterized, our understanding of most of these toxins is still insufficient. The generation rate, physicochemical properties, protein binding rate, biological toxicity, and relationship with the cardiovascular events of these toxins are all unclear. More research is needed to clarify the characteristics of various toxins and better identify patterns and correlations that may lead to novel therapeutic targets and interventions.

Finally, there is an urgent need to explore more effective strategies for clearing uremic toxins. Some therapeutic strategies, like adsorbents, have shown certain advantages in eliminating toxins, but they cannot yet be routinely applied in clinical practice. The efficacy and safety of these treatments still require further confirmation through more clinical studies.

In light of these considerations, the development of novel therapeutic strategies to target metabolic abnormalities is paramount. Interventions that not only address mineral imbalances but also mitigate the effects of uremic toxins could represent pivotal advancements in breaking the detrimental cycle linking CKD and CVD. Furthermore, the integration of multi-organ protective interventions may offer a synergistic approach to managing these interconnected conditions. Such strategies could encompass lifestyle modifications, pharmacological treatments, and innovative therapies aimed at restoring metabolic balance and protecting organ function.

In conclusion, the ongoing research into CKD and CVD highlights the necessity for a concerted effort across disciplines to deepen our understanding of the underlying mechanisms at play. By fostering collaboration among nephrologists, cardiologists, and researchers, we can pave the way for improved diagnostic tools and therapeutic options. Ultimately, addressing the intricacies of the CKD-CVD relationship will not only enhance patient outcomes but also contribute significantly to public health by reducing the burden of these prevalent diseases. Future studies must prioritize this integrative approach, focusing on both the biological and clinical aspects of CKD and CVD to develop targeted interventions that can effectively disrupt the vicious cycle and promote better health for affected individuals.

## Figures and Tables

**Table 1:** Summary of uremic toxin properties.

Uremic Toxin	Structure	Size (MW)	Protein Binding	Origin
Indoxyl sulfate	C_8_H_7_NO_4_S	213.2	86–98% bound to albumin	Dietary tryptophan
p-Cresyl sulfate	C_7_H_8_O_4_S	188.2	91–95% bound to albumin	Dietary tyrosine and phenylalanine
Trimethylamine N-oxide	C_3_H_9_NO	75.1	Free water-soluble	Dietary phosphatidylcholine, choline, betaine, and L-carnitine
Asymmetric dimethylarginine	C_8_H_18_N_4_O_2_	202.3	30% bound to albumin	Non-proteinogenic amino acid synthesized through post-translational methylation of arginine
